# A Systematic Review and Single-Arm Meta-Analysis on the Efficacy of High-Intensity, Focused Ultrasound for Benign Prostatic Hyperplasia Treatment: A Forgotten Option?

**DOI:** 10.7759/cureus.65384

**Published:** 2024-07-25

**Authors:** Carlos A Garcia-Becerra, Veronica Soltero-Molinar, Maria I Arias-Gallardo, Jesus E Juarez-Garcia, Natalia Garcia, Leonardo Fernández-Avila, Carlos M Garcia-Gutierrez

**Affiliations:** 1 Urology, Urovallarta Medical Center, Puerto Vallarta, MEX; 2 Anatomy, Universidad Autonoma de Guadalajara, Guadalajara, MEX; 3 Immunology, Centro de Investigación Biomédica de Occidente, Instituto Mexicano del Seguro Social, Guadalajara, MEX

**Keywords:** lower urinary tract symptom, ablation technique, high-intensity focused ultrasound ablation, prostatic adenoma, benign prostatic hyperplasia

## Abstract

Benign prostatic hyperplasia (BPH) is a non-cancerous enlargement of prostate tissue, commonly affecting older men. This condition leads to lower urinary tract symptoms (LUTS), which significantly affect the quality of life. Over time, extensive research has been conducted regarding BPH treatment, exploring various treatment options. High-intensity focused ultrasound (HIFU) is a non-invasive treatment modality that has shown promise in initial studies. However, evidence regarding its long-term efficacy and safety remains inconclusive. This study evaluates HIFU's safety and efficacy for BPH treatment, identifying gaps for future research. The study conducted comprehensive searches across the PubMed, Google Scholar, Cochrane Central, and ClinicalTrials.gov databases, covering English-language articles from 1994 to 2023. Inclusion criteria focused on peer-reviewed studies, with more than 10 patients utilizing ultrasound image-guided HIFU for BPH while excluding other HIFU modalities lacking ultrasound image guidance. Data extraction targeted primary outcomes (peak flow rate, International Prostate Symptom Score (IPSS), postvoid residual volume) and secondary outcomes (treatment time, follow-up duration). Statistical analysis utilized a random effects model with heterogeneity assessed by I² statistics and the Q test, alongside subgroup analysis based on study design. The risk of bias assessment employed the Cochrane Collaboration tool for randomized controlled trials and the methodological index for nonrandomized studies. Among 560 identified articles, 12 studies with 522 patients met the inclusion criteria. Primary outcomes showed improvements in Qmax (1 month: 2.50 ml/s, 12 months: 6.22 ml/s) and IPSS (1 month: -9.37 points, 12 months: -11.60 points). Reported complications included transient hematuria, hematospermia, and urinary retention. HIFU presents significant clinical improvements in treating BPH, albeit with slow progression attributed to specific techniques and the ablative approach. Manageable complication profiles are observed, yet study design flaws hinder a comprehensive evaluation of HIFU efficacy. The authors suggest areas for clinical optimization, emphasizing the necessity of further research.

## Introduction and background

Benign prostatic hyperplasia (BPH) is a common male condition. It is marked by a non-cancerous enlargement of prostate tissue surrounding the urethra, which ultimately narrows the urethral passage, leading to lower urinary tract symptoms (LUTS) [1]. BPH significantly affects the quality of life through symptoms such as urinary frequency and urgency, nocturia, and a weak urinary stream, leading to discomfort and interrupted sleep, and they may also result in complications such as bladder stones and urinary tract infections. The prevalence increases proportionally with age, and approximately one in every four men will suffer symptoms to some degree [1,2].

BPH is commonly treated with a combination of medications, such as alpha-blockers and 5-alpha reductase inhibitors, which are often the first line of treatment. For patients who have not responded to medical management or who have complications from bladder outlet obstruction due to BPH, minimally invasive procedures can be used. These include transurethral microwave thermotherapy and transurethral needle ablation, which target excess prostate tissue using thermal or radiofrequency energy [3].

High-intensity focused ultrasound (HIFU) has emerged as a promising therapeutic modality for tissue ablation since the 1950s [4]. HIFU seeks to deliver focused ultrasound waves to the tissue, inducing two main phenomena [5,6]: coagulative necrosis and acoustic cavitation. The first occurs because the focal points increase the temperature up to 60-100°C; the latter, acoustic cavitation, consisting of acoustic pressure, induces the formation of air cavities that contribute to tissue ablation [5,6].

Despite the numerous published studies on different aspects of HIFU for prostate cancer, the available evidence about the efficacy and safety of HIFU in treating BPH has been scattered and inconclusive.

The present study used a systematic review and meta-analysis to summarize and evaluate the evidence available for this possible forgotten option in BPH management. By synthesizing the existing evidence, this study also included identifying possible knowledge gaps for possible future research fields to increase the generalizability of the clinical use of this treatment option or to develop alternative and more efficient methods to improve this technique.

## Review

Methodology

Search Strategy

Based on the Population, Intervention, Outcome, and Study Design (PICOS) approach, we defined the question used for starting the methodology of this study. We conducted a systematic review and meta-analysis using the Preferred Reporting Items for Systematic Reviews and Meta-Analyses (PRISMA) statement recommendations [7].

We utilized the PubMed, ScholarGoogle, Central Cochrane, and ClinicalTrials.gov registries. The search method included articles published in the English language, from January 1994 to January 2023. Using the Boolean search strings “BPH” AND “Transrectal High-Intensity Focused Ultrasound”, “Benign Prostatic Hyperplasia” AND “Transrectal High-Intensity Focused Ultrasound”, we used “BPH” AND “HIFU”, “Benign Prostatic Hyperplasia”, and “HIFU”, or only the terms depending on the database.

The systematic review was performed by two investigators (C.A.G.B. and V.S.M.), who independently and blinded to the other investigators’ decisions reviewed the studies. The initial review was performed by analyzing the title/abstract and possible duplications; if the initial article assessment met the inclusion-exclusion criteria, the article was chosen for complete analysis by another author (C.M.G.G.). A second assessment for possible duplication was subsequently performed, followed by data extraction. All disagreements were resolved by consensus.

This study was registered with the PROSPERO registry (CRD42024534819).

Eligibility Criteria

Inclusion criteria were peer-reviewed articles in the English language published from 1994 to 2023, clinical trials or observational studies with a sample size of >10 patients, using ultrasound image-guided HIFU for BPH treatment, and containing pre- and posttreatment follow-up parameters. Exclusion criteria were purely abstracts, brief comments, editorial letters, literature reviews, systematic reviews or meta-analyses, case reports, case series with a sample of < 10 patients, studies with participants who received HIFU treatment in other image guide modalities apart from transrectal ultrasound image guides or studies that did not have pre- or post follow-up evaluations.

Data Extraction and Outcome Measurements

Data extraction was performed by three investigators. Two investigators performed a conscientious reading and data extraction, whereas the third author verified the extracted data. Primary outcomes were pre- and posttreatment peak flow rate (Qmax), International Prostate Symptom Score (IPSS), postvoid residual volume (PVR), prostate volume, posttreatment catheterization time, and every reported complication. The secondary outcomes were treatment time and duration of the follow-up period. Additional information was the authors, publication year, study type, and pooled demographic data of each sample.

The data were extracted from a unique database using Microsoft Excel (Microsoft Corporation, Redmond, WA, US). All missed data were managed first by attempting to obtain it from the original authors; if this was not possible, a mathematical derivation was performed using a recommended method; if these two criteria were not possible, the article was excluded.

Study Quality and Bias Assessment

The assessment was accomplished by four investigators using the Cochrane Collaboration tool for assessing the risk of bias for clinical trials [8] and the Methodological Index for Nonrandomized Studies (MINORS) [9] for observational studies. The assessment was performed during the data extraction phase. All disagreements were resolved with a consensus.

Statistical Analysis

For statistical analysis, effect size estimation was performed using the mean difference, followed by an inverse variance estimation for study weight. A subsequent planned subgroup analysis was performed divided by study design, observational studies, and experimental studies (designated for this article also as clinical trials). Heterogeneity between subgroups was measured with a test of subgroup differences, and finally, a pooled effect analysis was performed.

All analyses were performed using the random effect model with the heterogeneity variance estimated by the restricted maximum likelihood (REML) method; the heterogeneity magnitude was measured using primarily the I2 statistic and complemented by Cochrane’s Q test. Because the studies presented a different follow-up period, the studies were also analyzed according to the time frame, performing a separate analysis for 1-month, 3-month, 6-month, and 12-month follow-ups of each variable.

The complications were analyzed and are reported as percentages of the total number of studies included in this meta-analysis.

All the statistical analyses were performed using RStudio v2023.12.1+402 software ( http://www.rstudio.com/) and the “Metafor” package version 4.6-0 (https://cran.r-project.org/web/packages/metafor/index.html).

Results

Study Selection and Characteristics

**Figure 1:**
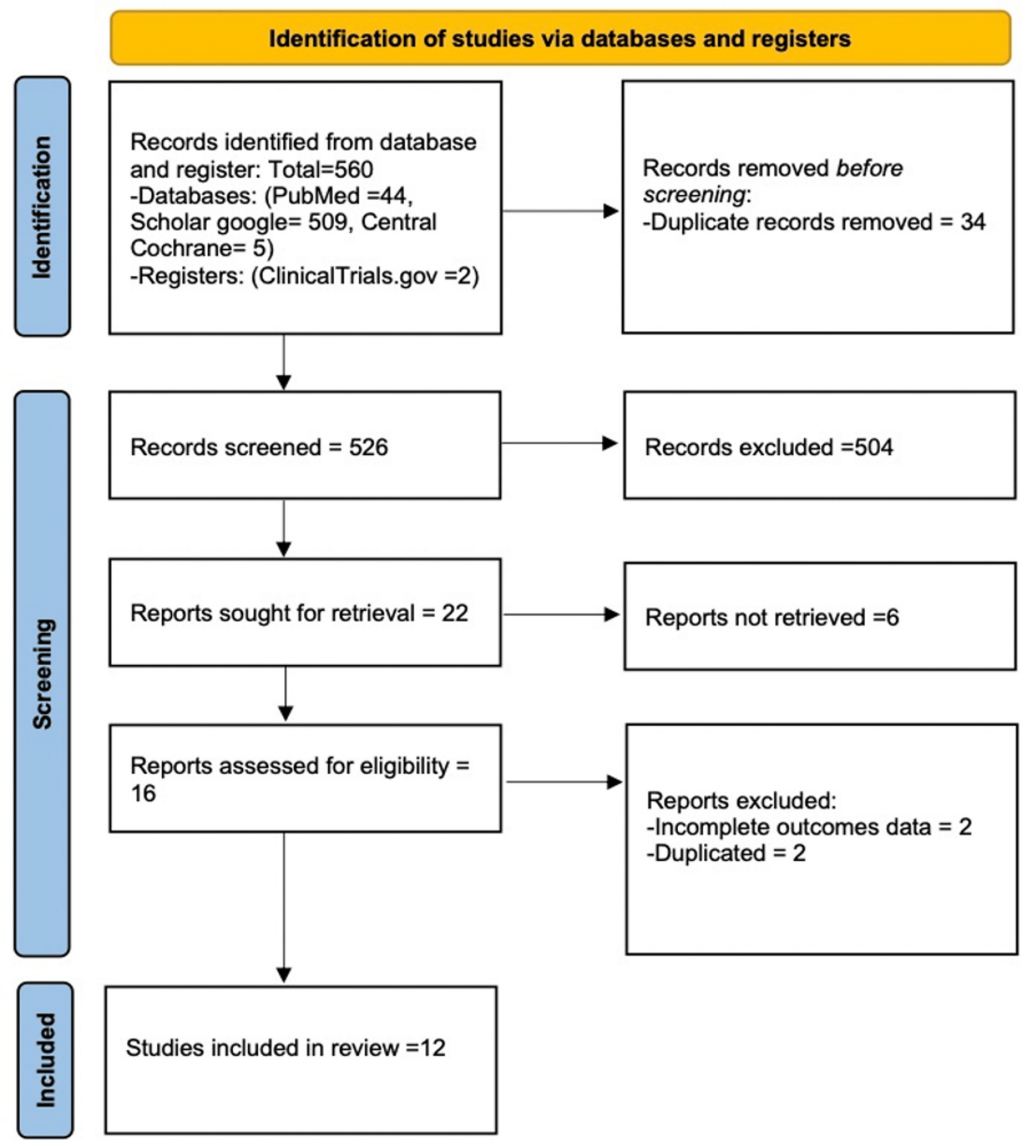
PRISMA 2020 flow diagram PRISMA: Preferred Reporting Items for Systematic Reviews and Meta-Analyses

Following the established systematic review methodology, a total of 560 articles were initially identified in databases or registries. After the complete screening, a total of 548 articles were excluded, leaving 12 eligible studies for inclusion. The complete selection process is shown in Figure 1. The 12 remaining eligible studies were 6 clinical trials and 6 observational studies, accounting for a total of 522 patients; both subgroups were single-arm studies without a control group. Some standard deviations were obtained from the Ranges using a formula reported in the literature [10]. Individual study information is presented in Table 1 [11-22].

**Table 1:** Individual characteristics of each study IPSS: International Prostate Symptom Score; PVR: postvoid residual volume

Author	Year	Sample Size	Follow-up	Mean Baseline Qmax	Mean Baseline IPSS	Mean Baseline PVR
Lu et al. [11]	2007	143	12 months	6 ml/s	24	75 ml
Uchida et al. [12]	1998	22	12 months	7.8 ml/s	19.7	57 ml
Mulligan et al. [13]	1997	13	24 months	9.9 ml/s	23	86.1 ml
Nakamura et al. [14]	1995	37	3 months	7.6 ml/s	23.6	79.1 ml
Sullivan et al. [15]	1997	20	3 months	9.1 ml/s	20.25	128 ml
Uchida et al. [16]	1995	28	6 months	8.8 ml/s	21.6	60 ml
Madersbacher et al. [17]	1994	50	12 months	8.9 ml/s	24.5	113 ml
Madersbacher et al. [18]	1997	102	12 months	9.1 ml/s	24.5	131 ml
Madersbacher et al. [19]	1996	30	6 months	8.8 ml/s	15.8	100 ml
Schatzl et al. [20]	2000	20	24 months	9.2 ml/s	14.7	94 ml
Ebert et al. [21]	1994	42	6 months	6.4 ml/s	17.8	205 ml
Bihrle et al. [22]	1994	15	3 months	9.3 ml/s	31.2	154 ml

Risk of Bias

The main risk of bias source detected using the designated tools for this study was the lack of randomization and blinding, which was derived from the lack of a control group in all the studies included. A summary of the complete assessment is provided in the Appendices.

Clinical outcomes

Qmax

The data analyzed for Qmax were available for all 12 studies for a total of 501 patients included. For subgroup analysis, a total of 232 patients were included in the observational studies, and 269 patients were included in the clinical trials.

The observational studies showed an effect size estimation at 1 month of 3.33 ml/s (95% CI=0.52, 7.20), including 2 studies (n=158), with Tau2=6.36 and I2=79.58%; at 3 months, 2 studies (n=41) had 5.44 ml/s (95% CI=2.87, 8.01), Tau2=0.92, and I2=23.87%; at 6 months, 4 studies (n=197) had 5.66 ml/s (95% CI=3.55, 7.77), Tau2=2.76, and I2=67.10%; and at 12 months, 3 studies (n=176) had 7.09 ml/s (95% CI=2.69, 11.48), Tau2=14.04, and I2=94.24%.

Accounting for the Clinical Trials subgroup, the effect sizes were estimated at 1 month and 1.70 ml/s (95% CI 1.46, 1.95; P=<0.0001); Tau2=0, and I2=0%; and 3 studies (n=115); at 3 months, 2.75 ml/s (95% CI 1.78, 3.72; P=<0.0001); Tau2=0.89 and I2=64.74%; and 6 studies (n=269); at 6 months, 3.53 ml/s (95% CI 2.76, 4.31; P=<0.0001); Tau2=0 and I2=0%, including 5 studies (n=232); and at 12 months, 5.12 ml/s (95% CI 3.10, 7.14; P=<0.0001) and Tau2=2.08 and I2=65.92%, including 3 studies (n=174).

The heterogeneity assessment with the subgroup difference analysis demonstrated an estimate of Tau2=0 and I2=0% and Q test P=0.4100 at 1 month; of Tau2=2.63 and I2=72.89% and Q test P=0.05 at 3 months; and of Tau2=1.60 and I2=70.88% and Q test P=0.06 at 6 months, and Tau2=0 and I2=0% and Q test P=0.42 at 12 months.

**Figure 2:**
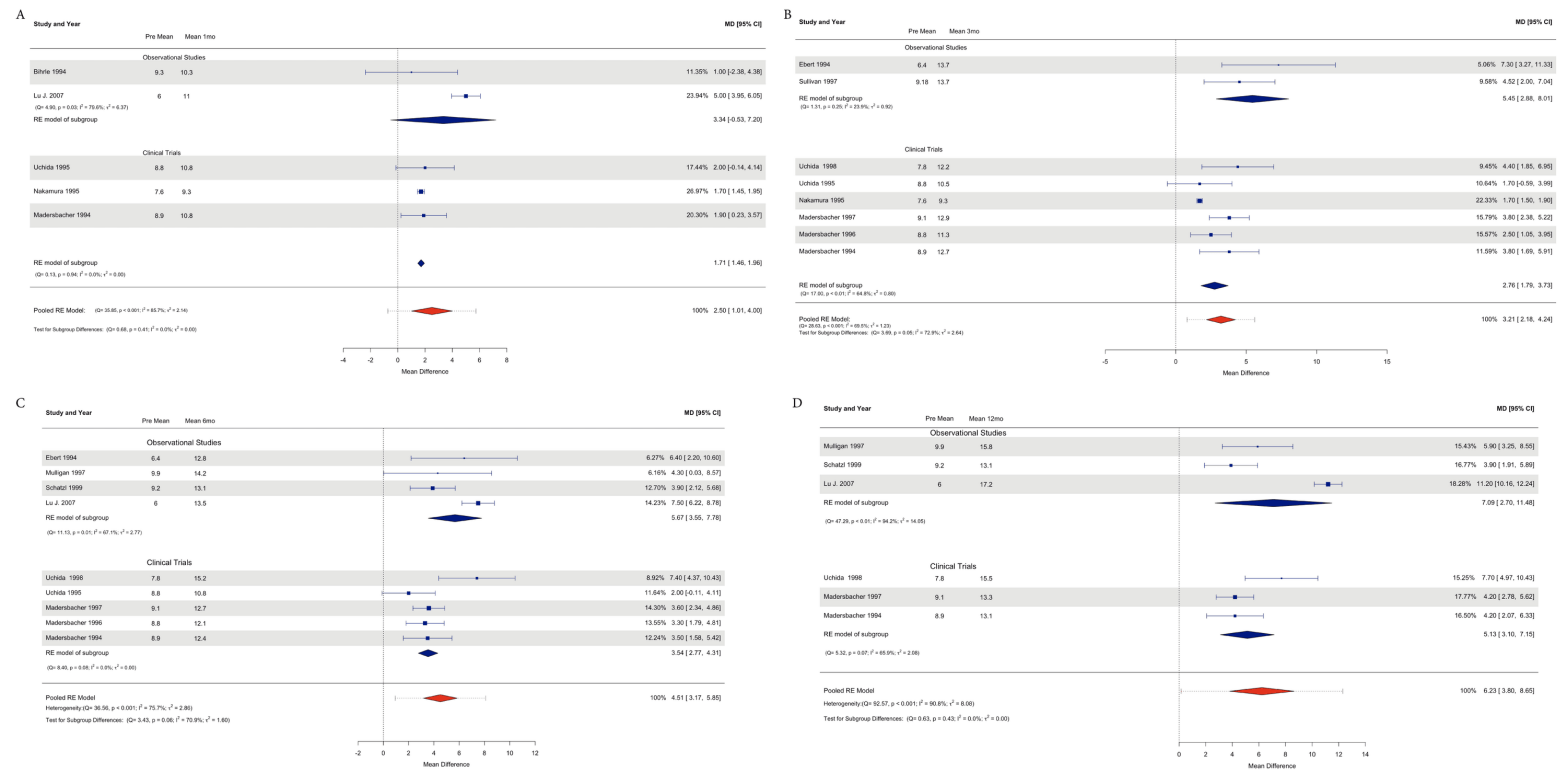
Forest plot with subgroup analysis and pooled analysis of the Qmax results A: Forest plot at 1-month follow-up; B: Forest plot at the 3-month follow-up; C: Forest plot at the 6-month follow-up; D: Forest plot at the 12-month follow-up [11-22]

The pooled effects for both subgroups were 2.50 ml/s at 1 month (95% CI=1.01, 3.99; P=0.001), 2.13 and I2=85.73%; 3 months, 3.20 ml/s (95% CI=2.17, 4.23; P=< 0.0001); Tau2=1.22, and I2=69.53%; 6 months, 4.51 ml/s (95% CI=3.17, 5.85; P=<0.0001); Tau2=2.86, and I2=75.68%; and 12 months, an effect estimated of 6.22 ml/s (95% CI=3.80, 8.64; P=<0.0001), Tau2=8.08, and I2=90.83% (Figures 2A-2D).

IPSS

IPSS data were obtained from all 12 studies; 497 patients were included, and the number of patients according to study type was 228 from observational studies and 269 from clinical trials.

An observational study analysis demonstrated an effect size estimation at 1 month to reduce the IPSS by -9.40 points (CI 95% -18.31, -0.48; P=0.03), including 2 studies (n=158) with Tau2=39.3 and I2=94.92%; at 3 months, we reduced the IPSS by -12.38 points (CI 95% -15.18, -9.58; P=< 0.0001), including 3 studies (n=52); at 6 months. We included 4 studies (n=193) to reduce the IPSS by -13.13 points (CI 95% -16.55, -9.48; P=<0.0001), including 3 studies (n=176); and at 6 months, we reduced the length by -15.14 points (CI 95% -19.66, -10.62; P=<0.0001), Tau2=14.69, and I2=92.82%.

The Clinical Trials subgroup showed an effect size estimate to reduce the IPSS: -9.93 points (95% CI 95% -10.97, -8.88; P=<0.0001) at 1 month with a Tau2=0.35 and I2=36.41%, including 3 studies (n=115); at 3 months to reduce it by -11.10 points (95% CI 95% -12.69, -9.51; P=<0.0001), with Tau2=2.25 and I2=81.21%, including 5 studies (n=241); at 6 months to reduce it by -10.56 points (95% CI -11.55, -9.57; P=<0.0001), with Tau2=0.07 and I2=5.08%; and at 12 months, with -11.60 (95% CI 95% -14.19, -9.00; P=<0.0001), with Tau2=3.99 and I2=84.29%, including 3 studies (n=174).

The subgroup difference analysis for heterogeneity showed an estimate of Tau2=0 and I2=0% and Q test P=0.01 at 1 month; of Tau2=0 and I2=0% and Q test P=0.43 at 3 months; of Tau2=1.44 and I2=43.62% and Q test P=0.18 at 6 months; and of Tau2=2.73 and I2=43.64% and Q test P=0.18 at 12 months.

**Figure 3:**
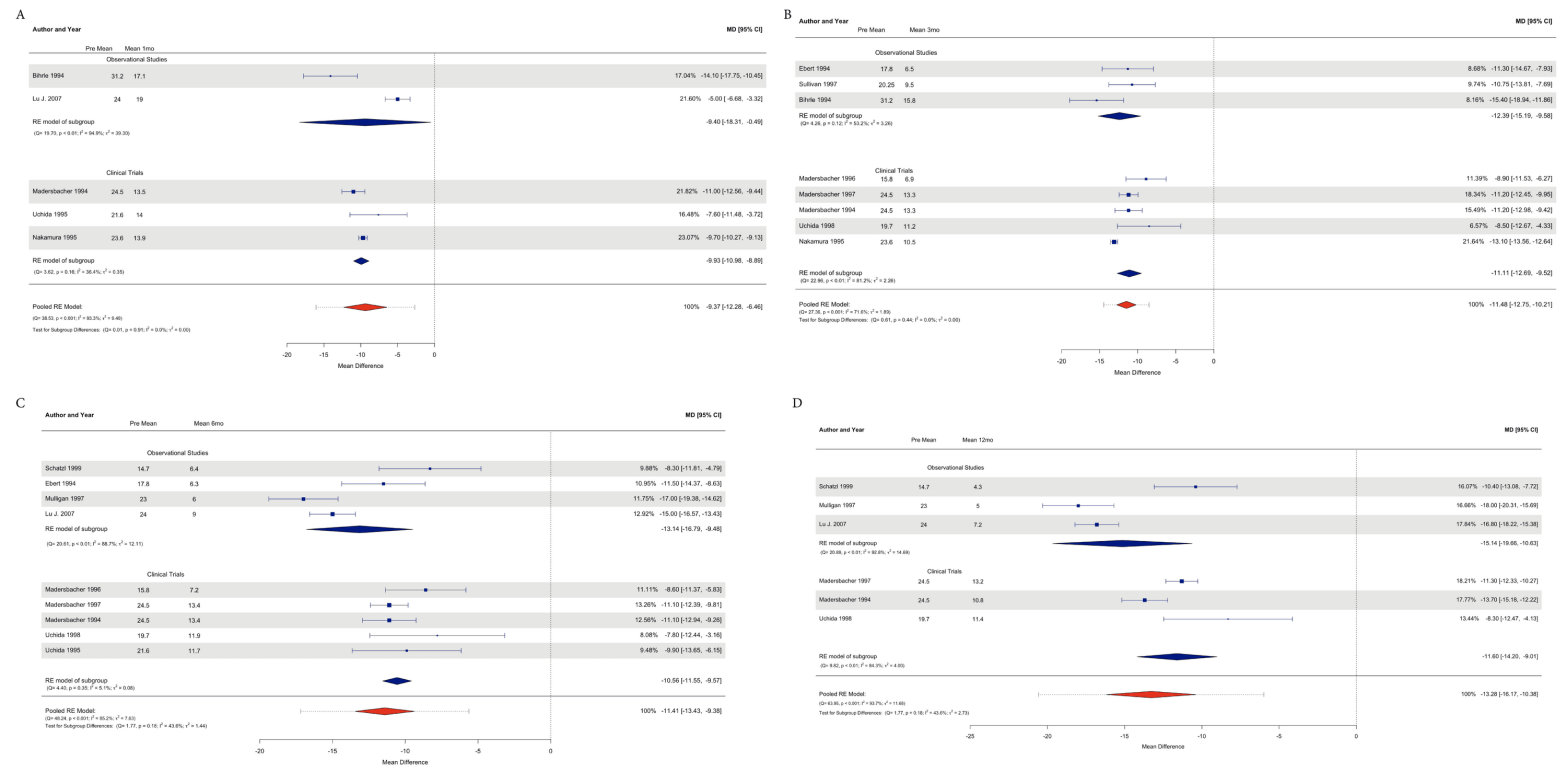
Forest plot with subgroup analysis and pooled analysis of IPSS results A: Forest plot at 1-month follow-up; B: Forest plot at the 3-month follow-up; C: Forest plot at the 6-month follow-up; D: Forest plot at the 12-month follow-up [11-22] IPSS: International Prostate Symptom Score

The pooled effects of all the studies were an estimate to reduce -9.37 points (95% CI -12.28, -6.46; P=0.001), Tau2=9.48 and I2=93.29%; at 3 months, -11.47 (95% CI -12.75, -10.20; P=<0.0001); Tau2=1.88 and I2=71.64%; at 6 months, -11.40 ml/s (95% CI -13.43, -9.37; P=<0.0001); Tau2=2.86 and I2=75.68%; and at 12 months, an effect estimated of -11.60 ml/s (95% CI -14.19, -9.00; P=<0.0001) Tau2=3.99 and I2=84.29% (Figures 3A-3D).

Post-Void Residual Volume

PVR data were obtained from all 12 studies with a total of 502 patients. The observational study group included 233 patients, and 269 patients were included in the clinical trial group.

The observational studies subgroup analysis for effect size estimation showed that at 1 month, the effect was reduced by -35.00 ml (95% CI: -38.67, -31.32; P=<0.0001) with Tau2=0 and I2=0%; at 3 months, the effect was reduced by -164 ml (95% CI: -223.98, -104.01; P=< 0.0001), Tau2=0 and I2=0%; at 6 months, it was reduced by -80.74 ml (95% CI: -154.06, -7.42; P=<0.03), Tau2=3877.33 and I2=97.49%; and at 12 months, it was reduced by -44.70 ml (95% CI: -47.50, -41.90; P=<0.0001), Tau2=0, and I2=0%.

Effect size estimates for clinical trials were as follows: reduced -26.34 points (95% CI -46.52, -6.16; P=<0.01) at 1 month with Tau2=227.27 and I2=72.87%; at 3 months, 42.22 points (95% CI -67.19, -17.26; P=0.0009); at 820.13 and I2=89.51%; at 6 months, -40.85 points (95% CI -65.78, -15.91; P=<0.0013); at Tau2=622.21 and I2=77.24%; and at 12 months, -60.71 (95% CI -101.20, -20.22; P=<0.0033), Tau2=1082.43, and I2=85.19%.

The subgroup difference test for heterogeneity assessment revealed the following results: Tau2=0, I2=0% and Q test P=0.40 at 1 month; Tau2=6864.87 and I2=92.59%; and Q test P=0.0002 at 3 months; Tau2=15.05 and I2=1.89%; and Q test P=0.31 at 6 months; Tau2=0 and I2=0%; and Q test P=0.43 at 12 months.

**Figure 4:**
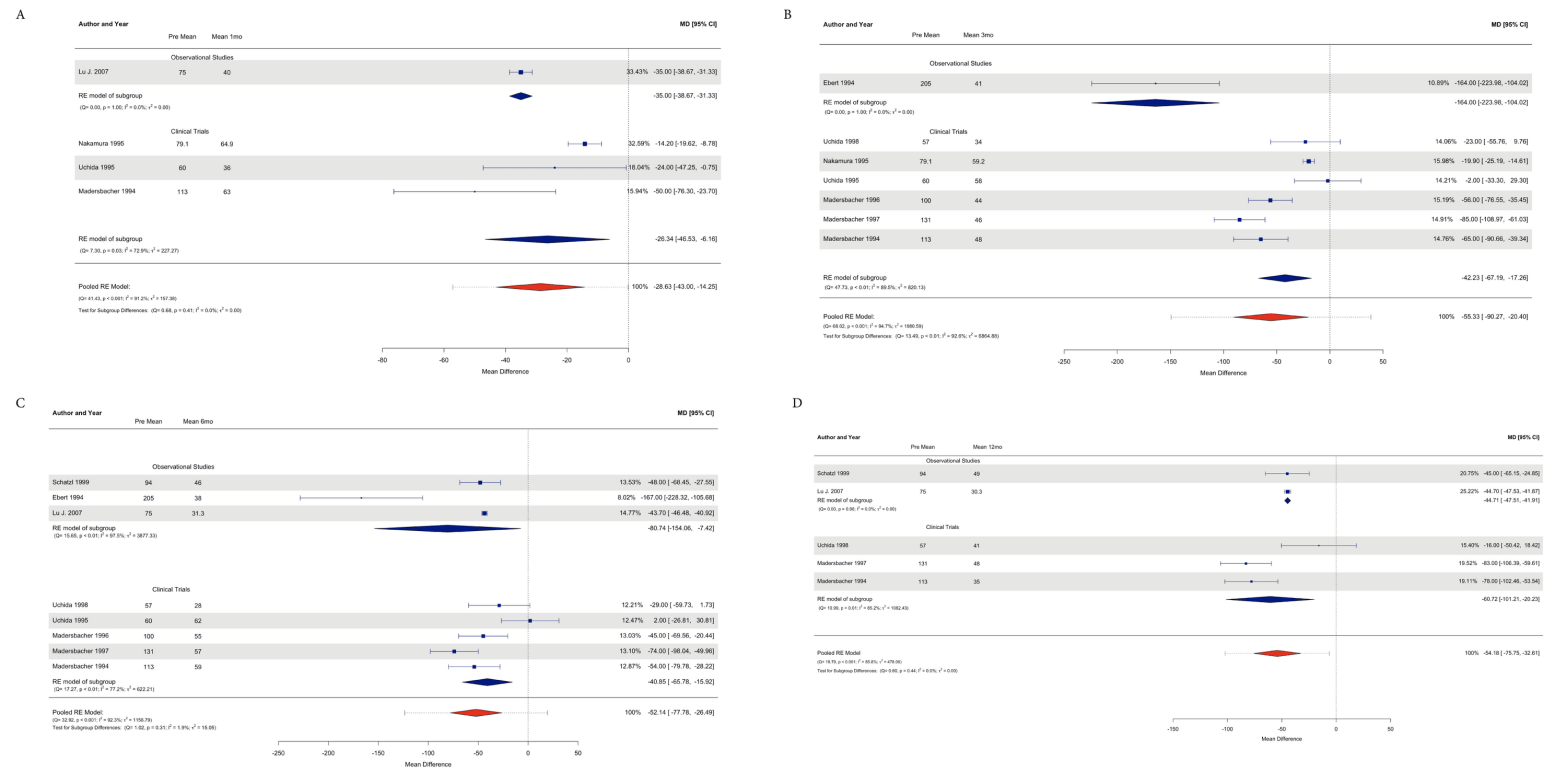
Forest plot with subgroup analysis and pooled analysis of PVR results A: Forest plot at one-month follow-up; B: Forest plot at the three-month follow-up; C: Forest plot at the six-month follow-up; D: Forest plot at the 12-month follow-up [11-22] PVR: postvoid residual volume

The pooled effects of PVR were as follows: -28.62 points (95% CI: -43.00, -14.25; P≤0.0001); Tau2=157.38; I2=91.24%; 3 months: -55.33 (95% CI: -90.26, -20.40; P=0.0019); Tau2=1980.58; I2=94.67%; 6 months: -52.13 ml/s (95% CI: -77.78, -26.49; P=<0.0001); Tau2=1156.79; I2=92.27%; and 12 months: an estimated effect of -54.18 ml/s (95% CI: 95% CI: -75, -32.61; P≤0.0001); and Tau2=478.05 and I2=85.80% (Figures 4A-4D).

Reported Complications

The reported complications were transient hematuria (THU), transient hematospermia (THS), transient urinary retention (TUR), posttreatment transurethral resection of the prostate (Pt-TURP) (due to poor clinical improvement), infections, enterovesical fistulas (EFs), and stenosis.

The percentages of complications reported by these studies were THU 58.33% (n=304), THS 58.33% (n=304), TUR 58.33% (n=304), Pt-TURP 50% (n=261), infections 25% (n=130), EF 16.66% (n=86), and stenosis 8.33% (n=43). The mean percentage and range of patients reported with each of these complications were 27.14% (range 97.62), 45.8% (range 92.9), 49.75% (range 77), 14.25% (range 15.11), 4.78% (range 4.44), 9.45% (range 17.1%), and 7.69%.

Treatment Time and Catheterization Time

Catheterization time and treatment time were completely reported in four studies: 8 ± 4, 4.8 ± 5.4, 4.3 ± 3.2, and 6 ± 10.25 days for catheterization time and 45 ± 12 min, 40.2 ± 14.1 min, 51.5 ± 22.3 min, and 48 ± 16.25 min for treatment time.

Discussion

According to the results of this systematic review and meta-analysis, HIFU demonstrated to be a treatment that produced a clinically significant improvement after BPH treatment; the primary outcomes within a period of 12 months of follow-up showed a Qmax improvement from a baseline of 3.20 ml/s at 3 months to 6.22 ml/s at 12 months and an IPSS reduction from baseline to -11.47 points at 3 months that then remained stable at 12 months, reflecting an important increase in quality of life. Her PVR decreased by -28.62 ml at 1 month and progressed to a reduction in volume of -54.18 ml at 12 months.

A prevalent side effect reported was transient urinary retention, which, according to Schatzl et al. [18], can be due to the slow necrotic tissue removal observed in these kinds of thermal therapies, suggesting that this factor could contribute to the reported decrease in Qmax. Madersbacher et al. hypothesized based on posttreatment cystoscopies that a possible explanation for the lack of a more significant clinical positive effect of HIFU for BPH treatment could be related to the technique that preserves the bladder neck, which could lead to a possible source of obstruction. He proposed that treatment in this specific region could have more positive results [23].

Although we found significant clinical improvement in primary outcomes and a relatively low percentage of severe, non-transient complications reported (EF, stenosis, or Pt-TURP), several limitations that made it challenging to perform an analysis and that could limit the conclusions were identified.

During the subgroup analysis of the different study designs (observational and experimental or clinical trials), no significant heterogeneity was found. Furthermore, during the pooled analysis, an important amount of heterogeneity was found, and we hypothesized that both groups of studies contained the same features that conditioned the heterogeneity of the effects. During the systematic review process, manuscripts with an observational and quasi-experimental study design were found, both without a control group. This situation prevented a subgroup analysis from revealing a between-group difference since the studies classified as experimental (clinical trials) could have similar limitations as observational studies and did not include a control group; thus, randomization or blinding was not included, leading to an increase in possible risk of bias and the subsequent presence of important effect heterogeneity.

## Conclusions

Based on the results of this systematic review and meta-analysis, we concluded that the available studies of HIFU for BPH treatment are limited in number and were limited in terms of proper design for good clinical evaluation. No randomized controlled trials were found, affecting the real estimation of the effect of this therapy. Moreover, HIFU is a safe option with a low number of complications reported and significant clinical improvement but with the limitation of slow clinical progression.

## Appendices

**Table 2:** Risk of bias assessment with the MINORS tool and the Cochrane Collaboration tool MINORS: Methodological Index for Nonrandomized Studies

Methodological Index for Non-randomized Studies (MINORS)						
Study Name:	Mulligan 1997 [13]	Bihrle 1994 [22]	Ebert 1994 [21]	Schatzl 2000 [20]	Lu 2007 [11]	Sullivan 1997 [15]
1. A clearly stated aim: the question addressed should be precise and relevant in the light of available literature	2	2	2	2	2	2
2. Inclusion of consecutive patients: all patients potentially fit for inclusion (satisfying the criteria for inclusion) have been included in the study during the study period (no exclusion or details about the reasons for exclusion)	2	2	2	2	2	2
3. Prospective collection of data: data were collected according to a protocol established before the beginning of the study	2	2	1	2	2	2
4. Endpoints appropriate to the aim of the study: unambiguous explanation of the criteria used to evaluate the main outcome which should be in accordance with the question addressed by the study. Also, the endpoints should be assessed on an intention-to-treat basis.	1	1	1	2	2	1
5. Unbiased assessment of the study endpoint: blind evaluation of objective endpoints and double-blind evaluation of subjective endpoints. Otherwise, the reasons for not blinding should be stated	1	1	1	1	1	1
6. Follow-up period appropriate to the aim of the study: the follow-up should be sufficiently long to allow the assessment of the main endpoint and possible adverse events	2	1	1	2	2	2
7. Loss to follow-up less than 5%: all patients should be included in the follow-up. Otherwise, the proportion lost to follow-up should not exceed the proportion experiencing the major endpoint	1	2	1	1	1	2
8. Prospective calculation of the study size: information on the size of detectable difference of interest with a calculation of 95% confidence interval, according to the expected incidence of the outcome event, and information about the level for statistical significance and estimates of power when comparing the outcomes Additional criteria in the case of comparative study	0	0	0	0	0	0
Additional criteria in the case of comparative study						
9. An adequate control group: having a gold standard diagnostic test or therapeutic intervention recognized as the optimal intervention according to the available published data	N/A	N/A	N/A	N/A	N/A	N/A
10. Contemporary groups: control and studied groups should be managed during the same time period (no historical comparison)	N/A	N/A	N/A	N/A	N/A	N/A
11. Baseline equivalence of groups: the groups should be similar regarding the criteria other than the studied endpoints. Absence of confounding factors that could bias the interpretation of the results	N/A	N/A	N/A	N/A	N/A	N/A
12. Adequate statistical analyses: whether the statistics were in accordance with the type of study with calculation of confidence intervals or relative risk	N/A	N/A	N/A	N/A	N/A	N/A
†The items are scored 0 (not reported), 1 (reported but inadequate), or 2 (reported and adequate). The global ideal score is 16 for non-comparative studies and 24 for comparative studies.						
Cochrane Collaboration’s tool for assessing risk of bias in randomized trials						
Study Name	Uchida 1998 [12]	Madersbacher 1994 [17]	Madersbacher 1997 [18]	Nakamura 1995 [14]	Uchida 1995 [16]	Madersbacher 1996 [19]
Items:						
Random Sequence Generation	SOME CONCERNS	SOME CONCERNS	SOME CONCERNS	SOME CONCERNS	SOME CONCERNS	SOME CONCERNS
Allocation Concealment	SOME CONCERNS	SOME CONCERNS	SOME CONCERNS	SOME CONCERNS	SOME CONCERNS	SOME CONCERNS
Blinding: participant and personnel	HIGH	HIGH	HIGH	HIGH	HIGH	HIGH
Outcome assessment	LOW	SOME CONCERNS	LOW	LOW	SOME	SOME
Incomplete outcome data (attrition bias)	SOME CONCERNS	LOW	LOW	LOW	HIGH	LOW
Selective reporting	LOW	SOME CONCERNS	LOW	LOW	SOME	LOW
Other bias	N/A	N/A	N/A	N/A	N/A	N/A

## References

[1] Lee SW, Chan EM, Lai YK (2017). The global burden of lower urinary tract symptoms suggestive of benign prostatic hyperplasia: A systematic review and meta-analysis. Sci Rep.

[2] Speakman M, Kirby R, Doyle S, Ioannou C (2015). Burden of male lower urinary tract symptoms (LUTS) suggestive of benign prostatic hyperplasia (BPH) - focus on the UK. BJU Int.

[3] Kim EH, Larson JA, Andriole GL (2016). Management of benign prostatic hyperplasia. Annu Rev Med.

[4] Maloney E, Hwang JH (2015). Emerging HIFU applications in cancer therapy. Int J Hyperthermia.

[5] Haar GT, Coussios C (2007). High intensity focused ultrasound: physical principles and devices. Int J Hyperthermia.

[6] Garcia-Gutierrez CM, Becerra-Herrejon H, Garcia-Becerra CA, Garcia-Becerra N (2022). High intensity focused ultrasound (HIFU) in prostate diseases (benign prostatic hyperplasia (BPH) and prostate cancer). Advances in Soft Tissue Tumors.

[7] Page MJ, Moher D, Bossuyt PM (2021). PRISMA 2020 explanation and elaboration: updated guidance and exemplars for reporting systematic reviews. BMJ.

[8] Higgins JP, Altman DG, Gøtzsche PC (2011). The Cochrane Collaboration's tool for assessing risk of bias in randomised trials. BMJ.

[9] Slim K, Nini E, Forestier D, Kwiatkowski F, Panis Y, Chipponi J (2003). Methodological index for non-randomized studies (minors): development and validation of a new instrument. ANZ J Surg.

[10] Hozo SP, Djulbegovic B, Hozo I (2005). Estimating the mean and variance from the median, range, and the size of a sample. BMC Med Res Methodol.

[11] Lü J, Hu W, Wang W (2007). Sonablate-500 transrectal high-intensity focused ultrasound (HIFU) for benign prostatic hyperplasia patients. J Huazhong Univ Sci Technolog Med Sci.

[12] Uchida T, Muramoto M, Kyunou H (1998). Clinical outcome of high-intensity focused ultrasound for treating benign prostatic hyperplasia: preliminary report. Urology.

[13] Mulligan ED, Lynch TH, Mulvin D, Greene D, Smith JM, Fitzpatrick JM (1997). High-intensity focused ultrasound in the treatment of benign prostatic hyperplasia. Br J Urol.

[14] Nakamura K, Baba S, Fukazawa R, Homma Y, Kawabe K, Aso Y, Tozaki H (1995). Treatment of benign prostatic hyperplasia with high intensity focused ultrasound: an initial clinical trial in Japan with magnetic resonance imaging of the treated area. Int J Urol.

[15] Sullivan LD, McLoughlin MG, Goldenberg LG, Gleave ME, Marich KW (1997). Early experience with high-intensity focused ultrasound for the treatment of benign prostatic hypertrophy. Br J Urol.

[16] Uchida T, Yokoyama E, Iwamura M (1995). High intensity focused ultrasound for benign prostatic hyperplasia. Int J Urol.

[17] Madersbacher S, Kratzik C, Susani M (1994). Tissue ablation in benign prostatic hyperplasia with high intensity focused ultrasound. J Urol.

[18] Madersbacher S, Klingler CH, Schatzl G, Schmidbauer CP, Marberger M (1996). The urodynamic impact of transrectal high-intensity focused ultrasound on bladder outflow obstruction. Eur Urol.

[19] Madersbacher S, Kratzik C, Marberger M (1997). Prostatic tissue ablation by transrectal high intensity focused ultrasound: histological impact and clinical application. Ultrason Sonochem.

[20] Schatzl G, Madersbacher S, Djavan B, Lang T, Marberger M (2000). Two-year results of transurethral resection of the prostate versus four 'less invasive' treatment options. Eur Urol.

[21] Ebert T, Graefen M, Miller S, Saddeler D, Schmitz-Dräger B, Ackermann R (1995). High-intensity focused ultrasound (HIFU) in the treatment of benign prostatic hyperplasia (BPH). Keio J Med.

[22] Bihrle R, Foster RS, Sanghvi NT, Donohue JP, Hood PJ (19941). High intensity focused ultrasound for the treatment of benign prostatic hyperplasia: early United States clinical experience. J Urol.

[23] Madersbacher S, Schatzl G, Djavan B, Stulnig T, Marberger M (2000). Long-term outcome of transrectal high-intensity focused ultrasound therapy for benign prostatic hyperplasia. Eur Urol.

